# Exploring tricine as a novel red cell cryopreservative: Lessons and future directions

**DOI:** 10.1111/vox.70088

**Published:** 2025-07-31

**Authors:** Thomas Bailey‐Schmidt, Christine V. Saunders, Chloë E. George, Thomas G. Scorer, Lynn M. R. McCallum, Tracey E. Madgett

**Affiliations:** ^1^ School of Biomedical Sciences, Faculty of Health University of Plymouth Plymouth UK; ^2^ Welsh Blood Service, Component Development and Research Laboratory Pontyclun UK; ^3^ University Hospitals Plymouth, NHS Trust Plymouth UK; ^4^ Centre of Defence Pathology, Royal Centre for Defence Medicine Birmingham UK

**Keywords:** blood processing, cryopreservation, cryoprotectants, red cell components, tricine

## Abstract

**Background and Objectives:**

Cryopreservation allows for storage of red blood cells (RBCs) beyond the standard 35‐day period. Current glycerol‐based methods are labour‐intensive and scale‐limited in application. Tricine has been identified as a potential alternative cryoprotectant (CPA), demonstrating efficacy in sheep RBC. This study aims to evaluate the biocompatibility and efficacy of tricine in human RBC cryopreservation.

**Materials and Methods:**

Human and sheep RBCs were exposed to varying concentrations of tricine (2.0–20.0% w/v) or glycerol (20.0–40.0% w/v). Biocompatibility was assessed via 24‐h incubation at 4°C, while cryoprotective efficacy was evaluated following freezing in liquid nitrogen, storage at −80°C and thawing at 37°C. RBC recovery was assessed via spectrophotometric estimation of haemolysis.

**Results:**

Tricine was biocompatible, with <1% haemolysis in both species. When frozen, tricine provided significant protection against cryoinjury in sheep RBC, with maximal recovery at 8.0% w/v (42.17% ± 10.96% of RBC recovered). However, tricine lacked cryopreservative efficacy in human RBC, with post‐thaw recovery rates on par with those seen following unprotected freezing. Even at the highest performing concentration (10.0% w/v), human RBC recovery remained low (16.08% ± 2.96%), highlighting the ineffectiveness of tricine in preserving human RBC integrity. Further analyses revealed greater hydrophilicity in sheep haemoglobin, which potentially influences freezing tolerance.

**Conclusion:**

Despite promising results within the ovine model, tricine lacks CPA efficacy for human RBC. Species differences in RBC physiology likely contribute to these discrepancies. These findings emphasize the need for rigorous model selection in cryopreservation research and further investigation into CPA mechanisms.

**Highlights**

- Tricine is biocompatible towards human red blood cells (RBCs) but ineffective in their cryopreservation.
- There remains a lack of evidence to support a reasonable hypothesis as to the mechanism by which tricine prevents cryoinjury in RBC.
- Tricine demonstrated significant cryoprotective effects in sheep RBC but not in human RBC, highlighting the importance of carefully selecting model systems for cryopreservation research.

## INTRODUCTION

Red cell concentrates (RCCs) are stored at 2–6°C and are viable for transfusion for 35 days post‐donation in the United Kingdom [1], due to progressive cellular deterioration in vitro. This shelf life presents challenges to ensuring effective levels of blood product are maintained in clinical areas of remote regions with infrequent access to blood services. While conservative prescribing and blood‐banking strategies can increase stock resilience and reduce waste, there remains a 1.5% annual rate of RCC wastage due to units time expiring [2].

Cryopreservation, or storage of biological materials at ultra‐low temperatures, offers a possible solution, allowing for the long‐term storage of RCC beyond their natural shelf life [3]. Currently, cryopreservation in the United Kingdom (through the National Frozen Blood Bank [NFBB], Liverpool) is utilized solely to maintain stocks of rare phenotype units for patients with complex phenotypes or antibody profiles. This ability to stockpile RCC without progressive deterioration in vitro has wider applications which would be advantageous to blood services. Frozen blood‐banking would facilitate retention of RCC stock ahead of periods where demand exceeds donor activity or when specific/regional events would otherwise place an unsustainable demand on the local donor pool (i.e., major sporting events, conflict, natural disasters). This relates to current concerns, with legislative guidance [4] encouraging ‘stockpiling’ of blood‐derived products to ensure resilience in times of shortage or increased demand. A frozen system would also increase accessibility to transfusion‐based care in areas that are isolated from blood services. Transporting frozen stock is logistically advantageous and would allow remote hospitals and clinics to maintain a supply of blood products without the need for regular replacement due to time expiry of stock [5].

**FIGURE 1:**
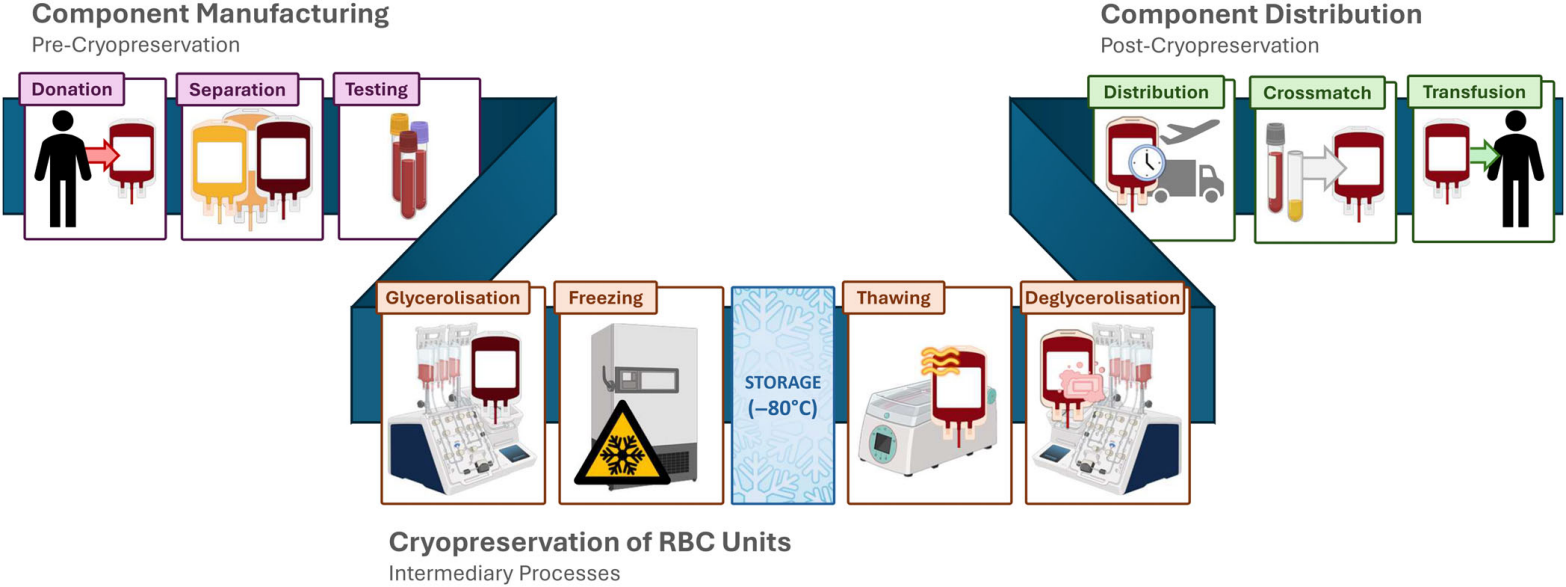
Current cryopreservation protocols fit within the ‘standard’ component journey of red cell concentrate stored at 4°C. Created in part using BioRender graphics tools (https://www.biorender.com/).

There are, however, constraints to cryopreservation that must be addressed before such methods can be employed at larger scale. Cryopreservation relies on the use of cellular cryoprotectants (CPAs) to prevent haemolysis due to dehydration/osmotic shock during freezing/thawing and the mechanical stresses of ice formation (collectively referred to as cryoinjury [6]). Current methods used by the NFBB utilize glycerol as the primary CPA for preservation of RCC units. While effective in its prevention of cryoinjury, efficacy of glycerol as a CPA is optimal at higher concentrations (20–40% w/v) and deglycerolizing units post‐thaw is complex and time‐consuming, combining liquid‐phase extraction and aphaeresis methods to remove glycerol from the red blood cells (RBCs) and produce a clinically transfusable product (Figure 1, [7]). These challenges to glycerol‐based methods have prompted research of alternative CPA, with the aim of improving current methods of unit‐scale cryopreservation (either by increasing post‐thaw cellular yield or streamlining the process of unit preparation) [8].

Recent work identified tricine (*N*‐tris(hydroxymethyl)methylglycine) as a possible alternative to glycerol [9]. Using rates of post‐thaw haemolysis as a measure of cellular recovery, Liu et al. [9] demonstrate tricine‐based methods provide significant protection against cryoinjury in sheep RBC, on par with glycerol and hydroxy ethyl starch (HES) methods. Their study also evaluated the quality of tricine‐cryopreserved RBC and reported that tricine‐based methods of cryopreservation yield RBC of greater functionality compared to those RBC frozen in glycerol or HES‐based media. This provides compelling evidence in favour of a tricine‐based method of RBC cryopreservation, pending confirmation within the human model.

While sheep RBCs have been used previously as a representative model in RBC studies [10, 11], the suitability of sheep RBC as proxy for human RBC in cryopreservative work has not been studied extensively. This study sought to determine if the positive effects of tricine on preserving sheep RBC could be transposed to cryopreservation of human RBC.

## MATERIALS AND METHODS

### Collection of RBCs

Sheep whole blood was obtained commercially from TCS Biosciences (Botolph Claydon, UK). Briefly, healthy sheep are bled into an equal volume of Alsever's solution to produce a final haematocrit (HCT) within 0.15–0.30 without further processing. All sheep RBCs were tested within 7 days of collection from donor.

Human donor‐derived RCCs were manufactured by the Welsh Blood Service from volunteer non‐renumerated whole blood donors with consent for collections to be used in research and tested within 7 days of donation. Ethical approval for the study was received from the University of Plymouth Faculty of Health Research Ethics and Integrity Committee (Reference: 4683).

In the text, we refer to cells and cell‐specific features as sheep and human RBC while describing the general model systems as ovine or human, respectively.

### Preparation of RBCs

For sheep RBC, any buffy coat was aspirated off during the first wash step, yielding a leucoreduced RCC. Human and sheep RBCs were prepared by washing thrice with 0.1 M phosphate buffered saline (PBS; pH 7.0, 0.1% sodium azide, Sussex Biologicals; Hailsham, UK) following centrifugation at 2000*g* for 10 min. All washing steps were isovolumetric and preserved the HCT of the donor product as determined by standard method [12].

### Cryopreservatives

#### Glycerol

Glycerol (99.8% purity, Merck Life Sciences UK Ltd.; Haverhill, UK) stocks were formulated in 0.1 M PBS such that the resultant concentration (% w/v) when added to the washed RBC component was either 20.0 or 40.0% w/v. pH was adjusted to 7.2 ± 0.1. Prepared stocks were autoclaved and stored at 4°C.

#### Tricine

Tricine (99% purity, Merck Life Sciences UK Ltd.; Haverhill, UK) stocks were formulated in 0.1 M PBS such that the resultant concentrations (% w/v) when added to the washed RBC component were 2.0, 4.0, 6.0, 8.0, 10.0, 15.0 or 20.0% w/v. pH was adjusted to 7.2 ± 0.1. Prepared stocks were autoclaved and stored at 4°C.

### Evaluation of cryopreservative biocompatibility

Washed RBCs from both human and sheep donors (50 μL, HCT 0.56 and 0.38, respectively) were added to 450 μL of prepared CPA and incubated at 22°C for 30 min. After induction, RBC/CPA suspensions were stored at 4°C (±2°C) for 24‐h before gentle resuspension. Sample supernatants were isolated by centrifugation (10 min at 2000*g*) and used to estimate the rate of haemolysis over the 24‐h period.

The following controls/standards were used and prepared for each biological replicate alongside treatment group(s):

1. *Negative treatment control*: Washed RBCs (50 μL) were suspended in 450 μL of PBS and stored at 4°C for 24‐h. Supernatant was isolated and cell‐free haemoglobin was measured as described.
2. *0% haemolysis standard*: Washed RBCs (50 μL) were suspended in 450 μL of PBS and incubated for 30 min at 22°C. Without any further incubation, the supernatant was isolated and cell‐free haemoglobin was measured as described.
3. *100% (complete) haemolysis standard*: Washed RBCs (50 μL) were suspended in 450 μL of deionized water (dH_2_O) and incubated for 30 min at 22°C. Complete haemolysis of the standard was ensured by pulse vortex mixing (6777 LSE™ Vortex Mixer; Corning Ltd.; Flintshire, UK), at the highest setting for 5 min. The supernatant/haemolysate was isolated and cell‐free haemoglobin was measured as described.

### Evaluation of CPA efficacy

Stock CPA was prepared as 450 μL aliquots in 2 mL vials suitable for immersion in liquid nitrogen (LN_2_). To each aliquot, 50 μL of washed RBCs were added and allowed to incubate at 22°C for 45 min. Samples were then flash frozen in LN_2_ for 30–40 s before storing at −80°C.

After 24‐h, the CPA/RBC samples were removed and thawed in a water bath (37°C) for 10 min. After RBCs were resuspended, the supernatant was isolated and used to estimate the rate of haemolysis.

The following controls/standards were used and prepared for each biological replicate alongside treatment groups:

1. *Negative treatment control*: Washed RBCs (50 μL) were suspended in 450 μL of PBS and stored at 4°C for 24 h following a 45‐min induction period at ambient (22°C) conditions. After 24 h, samples were warmed in a water bath (37°C) for 10 min prior to being resuspended and supernatant isolated.
2. *Positive treatment control*: Washed RBCs (50 μL) were suspended in 450 μL of PBS and inducted/frozen as described prior to storage at −80°C for 24 h. After which, samples were thawed in a water bath (37°C) for 10 min prior to being resuspended and supernatant isolated.
3. *0% and 100% haemolysis standards*: Prepared as described above.

### Estimation of haemolysis by spectrophotometric measure of cell‐free haemoglobin

Percent haemolysis was used to determine the rate of cellular recovery following cryopreservation or prolonged exposure to CPA, providing a quantitative measure for both biocompatibility and efficacy in preventing haemolysis due to cryoinjury.

Haemolysate was measured in the supernatant by direct spectrophotometry, adapted from established methodologies [12] for use in a 96‐well plate, where:

$$\%\text{Haemolysis}=\frac{{\mathrm{Abs}}_{\text{Sample}}-{\mathrm{Abs}}_{0\%\mathrm{Std}}}{A{\mathrm{bs}}_{100\%\mathrm{Std}}-{\mathrm{Abs}}_{0\%\mathrm{Std}}}*100.$$

The rate of RBC recovery for each sample was taken from the calculated rate of haemolysis:

%Cell recovery=1−%Haemolysis.

### Comparative analysis of haemoglobin protein surface hydropathy

#### Sequence alignment of haemoglobin subunit protein(s)

Amino acid (AA) sequences were obtained from the NCBI protein database (https://www.ncbi.nlm.nih.gov/; Table 1). Pairwise alignment (NCBI Blast Tool; [13]) was used to assign a position index to each residue and used as the basis for relative comparison of global and regional sequence hydropathy.

**TABLE 1:** Sequence information for haemoglobin subunits for human and sheep orthologues.

Species	Subunit	Accession ID	Length (AA)
Human (*Homo sapiens*)	Alpha	*NP_000508.1*	142
	Beta	*NP_000509.1*	147
Sheep (*Ovis aries*)	Alpha‐1	*XP_014959464.2*	142
	Beta	*NP_001091117.1*	145

*Note*: Reference/accession numbers are for the NCBI protein database (https://www.ncbi.nlm.nih.gov/).

#### Haemoglobin modelling and topology

A 3D model of human haemoglobin (HbA1) was produced in mol* modelling tool (https://molstar.org/; [14]); PDB files were accessed from NCBI (https://www.ncbi.nlm.nih.gov/) and used for protein modelling. Accessible surface area of AA residues on the resolved 4° structure was obtained from within mol*.

#### Hydrophobicity/hydrophilicity plots

The relative hydropathy between sheep and human haemoglobin orthologues was evaluated following methods and techniques described previously [15].

Residue hydropathy values were determined by applying an exponentially weighted variation model which considered the hydropathy of five adjacent residues. Relative hydropathy (with respect to human haemoglobin subunits) is taken as the difference in the weighted hydropathy scores of aligned residues between subunit sequences.

### Data analysis

Analysis of variance (ANOVA) was used to compare variances across the means of the control and treatment groups. Differences were considered significant between control and treatment groups where *p* values were less than 0.05. Statistical analysis was performed using GraphPad Prism (GraphPad Software, Boston, MA, USA, version 10.4.1(627)).

## RESULTS

### HCT of sheep and human RBC products

The HCT of human donor RBC after unit sampling/washing was 0.56 ± 0.09 (*n* = 9). Sheep RBCs were considerably more dilute and had a measured HCT of 0.38 ± 0.10 (*n* = 3).

### RBC biocompatibility of tricine‐based CPA

**FIGURE 2:**
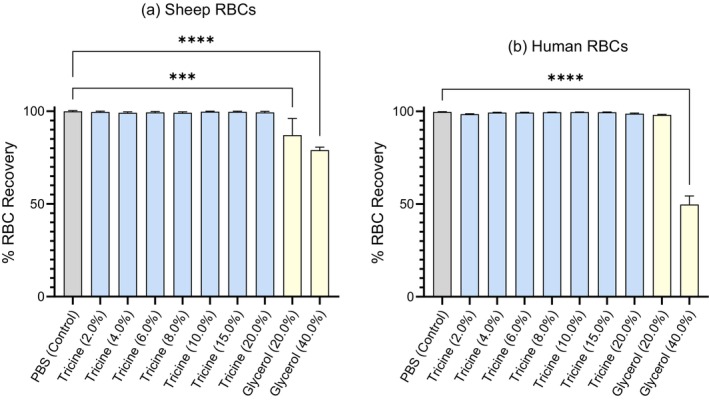
Percent (%) red blood cell (RBC) recovery following exposure of RBC to cryopreservative (CPA) media for 24 h at 4°C. CPA is reported in % w/v (i.e., grams per 100 mL) with unmodified phosphate buffered saline (PBS) (grey) as a solution control for tricine (blue) and glycerol (yellow) solutions. Results reported are mean values between biological replicates for (a) sheep RBC (*n* = 3) and (b) human RBC (*n* = 9) with bars indicating SD. Significance relative to control group as determined through analysis of variance (ANOVA) is indicated with respect to control values (*p ≤ 0.05; **p ≤ 0.005; ***p ≤ 0.0005; ****p < 0.0001).

In all concentrations of tricine examined (2.0%–20.0% w/v), red cell recovery was greater than or equal to 99.19% ± 0.43% in sheep (*n* = 3) and 99.33% ± 0.14% in human (*n* = 9) RBC (Figure 2a,b). Between glycerol concentrations, recovery increased from 78.98% ± 1.62% (40.0% w/v glycerol) to 87.05% ± 9.05% (20.0% w/v glycerol) in sheep RBC (*n* = 3) and increased from 49.74% ± 4.64% (40.0% w/v glycerol) to 98.03% ± 0.39% (20.0% w/v glycerol) in human RBC (*n* = 9).

### Efficacy of tricine‐based CPA in preventing haemolytic cryoinjury

**FIGURE 3:**
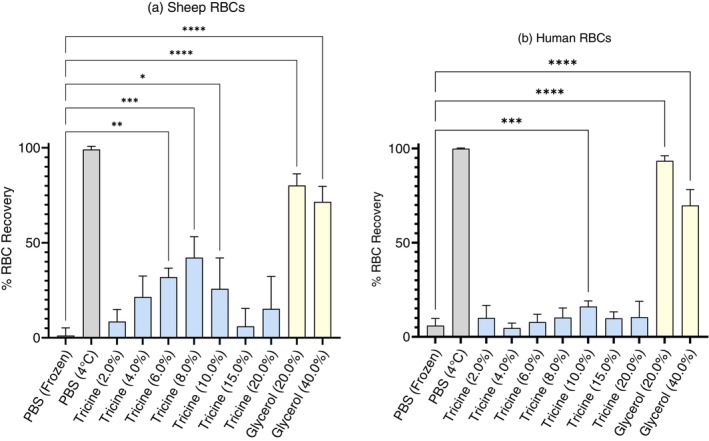
Percent (%) red blood cell (RBC) recovery following RBC/cryopreservative (CPA) media induction (incubation at 22°C for 45 min), flash freezing in liquid nitrogen, storage at −80°C (24 h) and subsequent thawing to 37°C. CPA is reported in % w/v (i.e., grams per 100 mL). Phosphate buffered saline (PBS) controls (grey) refer to unprotected freezing in PBS (frozen) and solution controls (incubated at 4°C) for cells frozen in tricine (blue) or glycerol (yellow) CPA. Results reported are mean values between biological replicates for (a) sheep RBC (*n* = 3) and (b) human RBC (*n* = 9) with bars indicating SD. Significance relative to frozen control group (PBS) as determined through analysis of variance (ANOVA) is indicated with respect to control values (*p ≤ 0.05; **p ≤ 0.005; ***p ≤ 0.0005; ****p < 0.0001).

In sheep RBC, tricine reduced red cell loss (Figure 3a) where CPA formulation was between 4.0 and 10.0% w/v tricine; maximal efficiency was seen in cells inducted in 8.0% w/v tricine with 42.17% ± 10.96% (*n* = 3) RBC recovery. When applied to human RBC however (Figure 3b), the maximal rate of recovery (16.08% ± 2.96%) is seen when cells were treated with 10.0% w/v tricine (*n* = 9; Figure 3b); this is the only concentration where a significant cryoprotective effect is observed in tricine‐supported treatment of human RBC. There is also a difference in rates of cell recovery between 40% and 20% w/v glycerol‐treated groups: in sheep RBC, recovery increased from 71.52% ± 8.16% (40% w/v) to 80.13% ± 6.14% (20% w/v) (*n* = 3) and increased from 69.71% ± 8.46% (40% w/v) to 93.40% ± 2.73% (20% w/v) in human RBC (*n* = 9).

### Effect of modifying the freezing/thawing process on the efficacy of tricine as a CPA

The effects of modified freezing or thawing on CPA efficacy were examined for the following CPA: 6.0% w/v tricine, 20.0% w/v glycerol and 40.0% w/v glycerol. We chose to move forward with 6.0% w/v tricine due to a previous study's [9] description of 6.0% w/v tricine as ‘optimal’ and our study finding no significant difference in CPA efficacy between 6.0% and 8.0% w/v tricine within the human model.

**FIGURE 4:**
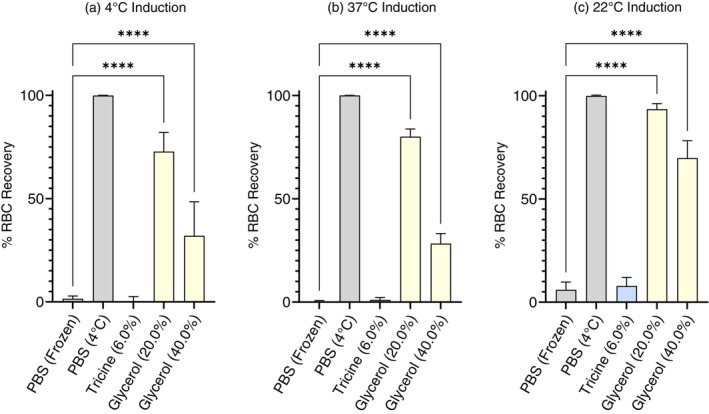
Percent (%) human red blood cell (RBC) recovery following RBC/cryopreservative (CPA) media induction for 45 min at (a) 4°C, (b) 37°C, or (c) 22°C, prior to flash freezing in liquid nitrogen, storage at −80°C (24 h) and subsequent thawing to 37°C. CPA is reported in % w/v (i.e., grams per 100 mL). Phosphate buffered saline (PBS) controls (grey) refer to unprotected freezing in PBS (frozen) and solution controls (incubated at 4°C) for cells frozen in tricine (blue) or glycerol (yellow) CPA. Results reported are mean values between biological replicates human RBC (*n* = 9) with bars indicating SD. Significance relative to control group as determined through analysis of variance (ANOVA) is indicated with respect to control values (*p ≤ 0.05; **p ≤ 0.005; ***p ≤ 0.0005; ****p < 0.0001).

Where human RBCs were incubated at 4°C (Figure 4a) or 37°C (Figure 4b) in 6.0% w/v tricine for 45 min, recovery following cryopreservation for 24 h was less than or equal to 1.0%, showing no significant effect of induction temperature on CPA efficacy nor improvement from rates of recovery seen where cells were inducted at 22°C prior to freezing (7.83% ± 4.20%, *n* = 9) (Figure 4c).

Induction at 4°C (Figure 4a) resulted in decreased cell recovery by 20.69% (20.0% w/v glycerol) and 37.73% (40.0% w/v glycerol) relative to rates of recovery where cells were inducted at 22°C (Figure 4c). Likewise, induction at 37°C (Figure 4b) resulted in a reduction in cell recovery by 13.37% (20.0% w/v glycerol) and 41.44% (40.0% w/v glycerol) when compared to rates of recovery of those cells inducted at 22°C (93.40% ± 2.73%, 20.0% w/v glycerol; 69.71% ± 8.46%, 40.0% w/v glycerol) (Figure 4c).

**FIGURE 5:**
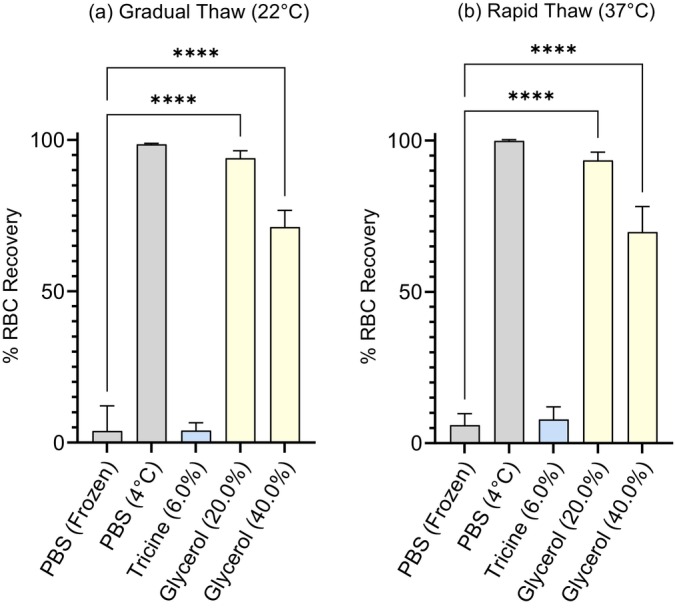
Percent (%) red blood cell (RBC) recovery following RBC/cryopreservative (CPA) media induction (incubation at 22°C for 45 min), flash freezing in liquid nitrogen, storage at −80°C (24 h) and subsequent thawing for (a) an hour at 22°C (gradual) or (b) 5 min at 37°C (rapid). CPA is reported in % w/v (i.e., grams per 100 mL). Phosphate buffered saline (PBS) controls (grey) refer to unprotected freezing in PBS (frozen) and solution controls (incubated at 4°C) for cells frozen in tricine (blue) or glycerol (yellow) CPA. Results reported are mean values between biological replicates for human RBC (*n* = 9) with bars indicating SD. Significance relative to control group as determined through analysis of variance (ANOVA) is indicated with respect to control values (*p ≤ 0.05; **p ≤ 0.005; ***p ≤ 0.0005; ****p < 0.0001).

Recovery of tricine‐treated RBC (6.0% w/v tricine) thawed at ambient temperature (22°C, over 45 min; Figure 5a) was not significantly different from the rate of recovery where RBCs (in 6.0% w/v tricine) were thawed at 37°C (3.96% ± 2.59%, *n* = 9; Figure 5b). Within glycerol‐treated groups, there was no statistical difference in rates of recovery between cells thawed rapidly (Figure 5b) and those cells thawed gradually (Figure 5a).

### Relative differences in subunit hydropathy between haemoglobin orthologues

Pairwise alignment of AA sequences identified non‐conserved AA residues between sheep/human orthologues of the alpha (19 sites) and beta (28 sites) subunits of haemoglobin, representing a 13% and 19% residue difference respectively, including similar and dissimilar AA substitutions.

**FIGURE 6:**
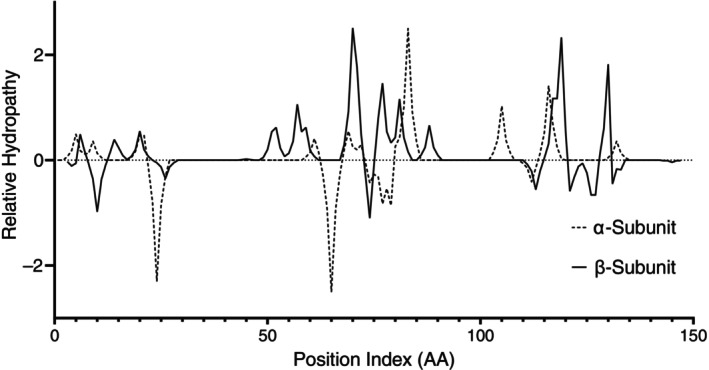
Relative hydropathy between alpha (dashed) and beta (solid) haemoglobin subunits of *Ovis aries* and *Homo sapiens*. Positive values indicate a more hydrophilic residue in the sheep orthologue and negative values indicate a more hydrophobic residue in the sheep orthologue. AA, amino acid.

Based on previously reported values [16], hydropathy scores between species indicate that human haemoglobin is marginally more hydrophobic in both alpha and beta subunits (0.4% and 4.6%, respectively) when compared to sheep haemoglobin. This indicates that most of the non‐conserved residues between the haemoglobin orthologues would produce a sheep haemoglobin tetramer (α_2_β_2_) with a greater hydrophilicity compared to the human orthologue (Figure 6).

**FIGURE 7:**
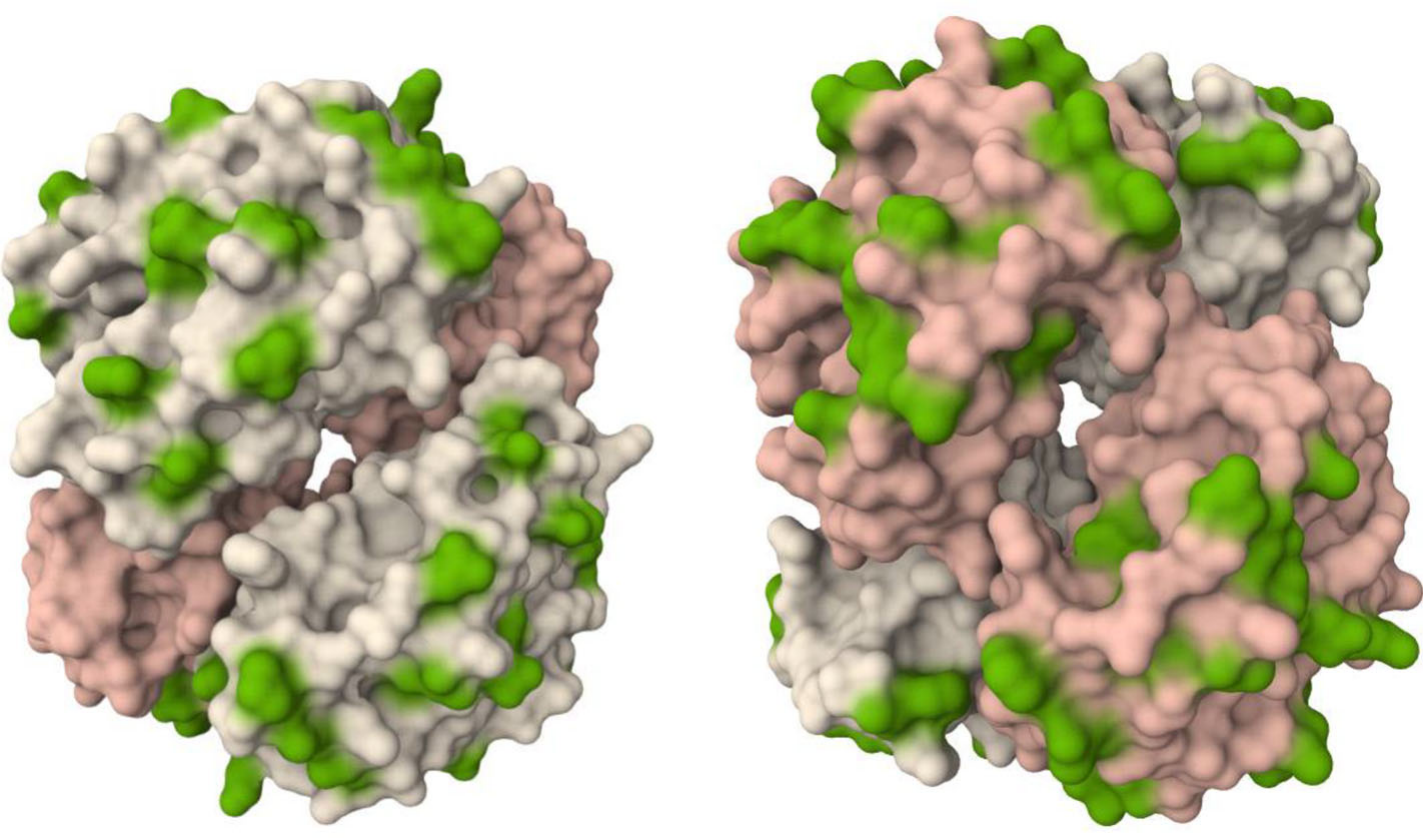
Resolved haemoglobin tetramer (α_2_β_2_) with non‐conserved sites between human and sheep orthologues highlighted in green. Images are 180° rotations of the same model about the *z*‐axis (vertically orientated) with subunit pairs coloured pink (alpha subunits) and white (beta subunits), with non‐conserved residues (between species orthologues) highlighted in green.

Where these non‐conserved residues are identified on a resolved haemoglobin model (Figure 7), we see most (45 out of 47; 95.7%) of the non‐conserved residues are arranged on the solvent‐facing surface of the tetramer.

## DISCUSSION

While the current study demonstrates tricine is biocompatible with human RBC in vitro, the study has established that tricine is ineffective as a CPA in human RBC. Having examined a broad range of concentrations of tricine (2.0–20.0% w/v) this study found no feasible concentration (as limited by tricine's solubility at ~25 g/100 mL) or means of treatment that enabled the prevention of cryoinjury in human RBC by tricine, despite previous [9] and current work demonstrating marginal efficacy in preventing cryoinjury in sheep RBC.

Rationale for the lack of efficacy of tricine as a CPA in human RBC cannot be offered by the methods and scope of the current study. However, we are able to draw from the literature and discuss tricine as a potential CPA based on its' previous and current usage. Tricine is a synthetic compound and was established as an applicant in protein and molecular biology following its review by Good et al. [17] as a hydrogen donor/buffer in biological media. Looking to expand the inventory of biological buffers available at the time, Good et al. [17] posed the ideal buffering agent would (i) have a pKa value between 6.0 and 8.0 (thus maintaining normal physiological pH), (ii) be highly soluble in aqueous solutions, (iii) be non‐permissible through cellular membranes, (iv) be non‐salt forming and (v) demonstrate dissociation/buffering behaviour independently of concentration or temperature. Considering the features of a freezing system (dynamic intermolecular bonding and solution compositions), Good's criteria describe relevant characteristics for a cryopreservative. A pKa value near typical cellular pH would maintain an environment within physiological tolerances and support cell homeostasis. Likewise, aqueous solubility and impassivity to salts, temperature or concentration would be advantageous in maintaining stability in a freezing/thawing system. However, there is little information available on the overlap between tricine's function as a biological molecule and RBC physiology and no specific evidence describing how tricine interacts with the RBC membrane or how it would behave as a CPA.

The ability of a CPA to cross the RBC membrane (either passively or actively) is of interest to cryobiologists. When considering CPAs that have been categorized ‘permeating’ or ‘non‐permeating’ by their ability to occupy the intracellular compartment, it is appreciated that the efficacy of the CPA is dependent on the occupation of the relevant cellular spaces. Glycerol, for example, is a permeating CPA and its mechanism of action is dependent on its occupation of both the intra‐ and extra‐cellular environment [18]. Given that tricine has been theorized to work similarly to glycerol due to shared structural motifs [9], the means (or lack thereof) by which tricine permeates the intracellular compartment is relevant to understanding its role as a CPA. To date, there has been no work to establish the mechanism and/or kinetics of tricine uptake by RBC.

When tricine was initially proposed as a CPA, Liu et al. drew on the similarities between the three hydroxyl groups of glycerol and the tri(hydroxymethyl) motif of tricine to propose a similar mechanism of action as a cryopreservative [9]. This is reasonable given the propensity of hydroxyl‐bearing compounds to form hydrogen bonds with solvent water and consensus among cryobiologists that such bonding disturbs the formation of crystalline ice and encourages preferential solvation of cellular proteins and membrane structures [19]. Work by Dashnau et al. [20] and Towey et al. [21] illustrates this point in their studies of hydrogen‐bonding networks within a glycerol/water solution, describing glycerol's ability to disrupt the hydrogen bonding of bulk water through the formation of solvation shells. Both studies cite glycerol's ability to rotationally conform around its propyl backbone as the key factor contributing to the intermolecular bonding seen in aqueous glycerol solutions [20, 21], a feature not yet established in tricine.

An unexpected outcome of this work has been the divergent results produced between RBC models regarding the efficacy of a tricine‐based system of cryopreservation. Given the ovine model's established role in both in vitro and in vivo translational research [22], key differences between sheep and human erythroid physiology were examined, particularly those with potential impact on cellular tolerance to CPA‐supported cryopreservation. Work by Simonova et al. provides a highly detailed analysis of the ovine model's use in transfusion research [22, 23]. Human RBCs are around 90 fL and three times larger than sheep RBCs (~30 fL). While sheep have slightly more RBC per volume of plasma [23], sheep typically present with a lower HCT (~0.3 L/L, HCT) compared to humans (~0.5 L/L) [23]. Given we made no attempt to correct the HCT between species, the difference in HCT was conserved within the RBC/CPA suspension. Despite differences in cellular dimensions, sheep RBCs have a comparable mean corpuscular haemoglobin concentration (MCHC) (~328 g/L) to humans (~340 g/L) [23]. The significance of HCT and MCHC regarding cellular tolerance to cryoinjury has not been studied directly, but indications of their effect can be derived from previous work [23, 24].

Studies by Mazur and Rigopoulos [24] and Mazur et al. [25] investigate solute hyper‐concentration in freezing systems contributing to freezing injury. They describe how cells become trapped in persistent liquid‐phase regions by ice‐lattice structures. Mazur et al. [25] term these regions the system's ‘unfrozen fraction’ and identify a critical threshold: RBC survival drops from over 80% at an unfrozen fraction of 0.14 to less than 20% when reduced below 0.07. Given the difference in RBC dimensions and density (HCT) between human and sheep, it could be posed that smaller, more diffuse RBC (i.e., sheep) would exhibit greater tolerance to ice‐mediated injury. A replicate study where initial RBC products were made to a standard HCT would allow for the role of cell size and density to be determined by inter‐ and intra‐specific comparison.

Examining haemoglobin orthologues identifies a potential role of haemoglobin in RBC freeze‐tolerance. Our study has established the sheep haemoglobin orthologue is more hydrophilic than the human equivalent by virtue of non‐conserved AA residues. Similar work has been done historically in other species by Bogner et al. [15, 26] alongside comparisons of inter‐species differences in erythrocyte water content and osmotic tolerances. Bogner et al. [15] establish a correlation between cellular water content and haemoglobin hydropathy with greater hydrophilicity indicating a reduced RBC water content (*r* = 0.830). While Bogner et al. [15] did not include the ovine model in their original analysis, their data on sheep RBC water content paired with our hydropathy data are congruent with previous observations [15]. The relationship between haemoglobin hydropathy and freezing tolerance becomes apparent when examining the relationship between haemoglobin hydropathy and the osmotically unresponsive water fraction between human and camel RBC (haemoglobin of the latter being more hydrophilic). Bogner et al. [26] report that in human RBC 16% of all intracellular water is osmotically unresponsive, where that fraction increases to 66% in camel RBC.

Bogner et al. [15] establish that membrane diffusion and ion/water transport differences between species are insufficient to explain the difference in tolerance to osmotic dehydration between species and propose a relationship between haemoglobin hydration and RBC tolerance to osmotic stressors. This relationship would describe sheep RBC exhibiting tolerance to dehydration by virtue of the hydrophilicity of haemoglobin. While previous work [15] does not consider freezing stress, nor report the osmotic‐responsive water fractions of sheep RBC, reasonable extrapolation can be made given freezing is effectively a dehydrating process with water removed by sequestration into ice rather than being lost through evaporation.

Our study confirms the biocompatibility of tricine with human RBC but demonstrates its lack of efficacy as a CPA. While tricine prevents cryoinjury in the ovine model, its mechanism of action does not translate to human RBC. These differences highlight species‐specific differences in cellular tolerance to cryopreservation and suggest an essential relationship between CPA and cellular physiology for effective cryopreservation. These findings underscore the importance of carefully selecting models for cryopreservation research and suggest further study of CPA mechanisms is needed to develop effective methods of RBC cryopreservation.

## CONFLICT OF INTEREST STATEMENT

The authors declare no conflicts of interest.

## Data Availability

The data that support the findings of this study are available from the corresponding author upon reasonable request.
